# Risk of temperature, humidity and concentrations of air pollutants on the hospitalization of AECOPD

**DOI:** 10.1371/journal.pone.0225307

**Published:** 2019-11-26

**Authors:** Cai Chen, Xuejian Liu, Xianfeng Wang, Wei Li, Wenxiu Qu, Leilei Dong, Xiyuan Li, Zhiqing Rui, Xueqing Yang

**Affiliations:** 1 Biomedical Engineering Institute, School of Control Science and Engineering, Shandong University, Jinan, China; 2 The First General Internal Medicine, Shengjing Hospital, China Medical University, Shenbei New District, Shenyang, Liaoning Province, China; 3 Department of Ecology and Environment of the People’s Republic of Shandong, Jinan, China; 4 Institute of Software, Chinese Academy of Sciences, Zhong Guan Cun, Beijing, P. R. China; 5 Helmholz Centre for Environmental Research (UFZ), Department of Bioenergy, Leipzig, Germany; The Ohio State University, UNITED STATES

## Abstract

**Aim:**

To investigate the effect of temperature, humidity and the concentration of ambient air pollution on the hospitalization of AECOPD.

**Method:**

Hospitalization record was obtained from Shenyang Medical Insurance Bureau, concluding patient’s age, gender, income hospital time, outcome hospital; Generalized additive model was used to analyze the relationship between temperature, humidity, the concentration of ambient air pollution and the hospitalization of AECOPD.

**Result:**

The effect of ozone on admission rate in male group was higher than that in female group. Ambient air pollution had a weak influence on age≤50 group. It was found that the optimal lag day for daily relative 40 humidity to age≤50 group, 50<age≤60, 60<age≤70 group and age>70 group was on lag5, lag4, lag4 and lag5, respectively.

**Conclusion:**

Air pollution, relative humidity and temperature can increase the risk of admission for acute exacerbation of COPD, and in this process there was a lag effect.

## Introduction

Environmental problems has become one of public problems in the world, ambient air pollution has been put closer attention to, which has aroused public’s health and environmental awareness. More epidemiological investigations have proved that ambient air pollution such as particulate matter (PM_2.5_ and PM_10_), sulfur dioxide (SO_2_), nitrogen dioxide (NO_2_) and ozone(O_3_), can cause acute inflammatory injury of respiratory tract and exacerbate pulmonary chronic diseases[1–3]. One study showed that fine particulate matter (PM_2.5_) with oxidized organic material was associated with a greater inflammatory response, which increased the amount of neutrophil and the level of CXCL-1 and TNF-α protein[4]. The published research proved that after the particulate matter (PM) was engulfed by alveolar macrophages, the airway inflammatory mediators increased, such as interleukin (IL)-6 and IL-18, which could lead to more inflammatory cell infiltration and airway mucus secretion[5]. A study showed that when Sprague-Dawley rats exposed to SO_2_ acutely, acute inflammation with neutrophil and macrophage airway infiltration was presented and inflammatory infiltrates remained in lung tissue for at least 14 days[6]. Previous research has established that NO2 exposure could induce pulmonary inflammatory response, mucus formation, and Th1/Th2 imbalance in control group[7]. It was reported that sex differences in acute O3-induced airway physiology responses and airspace influx without significant difference in other injury and inflammation measures[8].

Another research has demonstrated that per 10ug/m3 increase for SO_2_ was linked to -3.37 (95% confidence interval, CI, -5.39- -1.30) percent variation for forced expiratory volume after one second[9]

Furthermore, published reports have shown that meteorological factors such as temperature and barometric pressure, have a negative influence on respiratory diseases[10,11]. A study conducted in Hong Kong demonstrated that per 1°C increase in wintertime was associated with 1.20% (95% CI, 1.08–1.32) increase for total respiratory diseases hospitalization and 1.41% (95% CI, 1.15–1.71) increase for COPD hospitalization[12].

Shenyang, located in northeast China (123.38E, 41.8N), is the economic, cultural and transportation center in northeast China, which has suffered from severe air pollution for many years. In recent years, the minimum temperature in Shenyang Winter was below -20 degrees Celsius and the maximum temperature in summer was over 35 degrees Celsius. This paper purposed to estimate the effect of ambient air pollution and meteorological factors on AECOPD hospitalization in Shenyang.

## Materials and methods

Hospitalization record was obtained from Shenyang Medical Insurance Bureau, concluding patient’s age, gender, income hospital time, outcome hospital, work place and residence place. All this hospitalization data was only used to explore the effect of air pollution on AECOPD. And we had signed a confidentiality agreement, and this part of the data will not be disclosed. Fine particulate matter (PM_2.5_), sulfur dioxide (SO_2_), nitrogen dioxide (NO_2_), ozone (O_3_) and inhalable particle (PM_10_) were collected from Shenyang Environmental Protective Bureau. Daily average temperature, daily average wind speed and daily average relative humidity were attained from Shenyang Meteorology Bureau.

In this paper, generalized additive model with the link function of Poisson distribution was used to estimate the influence of air pollution on relative risk (RR) of hospitalization for acute exacerbations of chronic obstructive pulmonary disease (AECOPD)[13]. Patients who met the following criteria were included: (1) resided and worked in the study area during study period and (2) were admitted for AECOPD. People who met the following were excluded (1) not resided and worked in Shenyang during study period; (2)patients with duplicate records; and (3) patients admission within more than once among a week.

Stratification analysis based on age was divided to four group (age≤50 group, 50<age≤60 group, 60<age≤70 group and age>70 group), considering the sensitivity and tolerance to different group for temperature change, humidity change and air pollution were different. Stratification analysis based on gender was divided to two group: male group and female group.

$$\begin{array}{l} \text{Log}\left[\text{E}\left(y_{t}\right)]\right]=\alpha +\beta_{1}x_{i}+ns\left(Temperature,3\right)+ns\left(Humidity,3\right)+\\ ns\left(Wind,5\right)+ns\left(Time,4\right)+ns\left(Pressure,5\right)+\beta_{2}factor\left(DOW\right)+\\ \beta_{2}factor\left(Holidays\right) \end{array}$$ (1)

where α is the intercept value; β_1_, β_2_ and β_3_ are regression correlations; *E*[(*y*_*t*_)] represents the expected number of daily hospital admissions at day t; *x*_*i*_ is the daily air pollutant concentration; Temperature is daily average temperature; Humidity is the daily relative humidity; Pressure is the daily average pressure; Wind is the daily average wind speed; DOW is a dummy variable for the day of the week; *ns* represents a smoothed function[14,15]. Results are displayed as percentage change in relative risk (RR) of hospitalization and its 95% confidence intervals (CI) for a 10 μg/m^3^ increase in daily ambient air pollution concentration. Furthermore, we investigate the relationship between AECOPD and daily average temperature and relative humidity. Where *X*_*i*_ represents daily average temperature and relative humidity. (2)

$$\begin{array}{l} \text{Log}\left[\text{E}\left(y_{t}\right)\right]=\\ \alpha +\beta_{1}X_{i}+ns\left(Wind,5\right)+ns\left(Time,4\right)+ns\left(Pressure,5\right)+\\ \beta_{2}factor\left(DOW\right)+\beta_{3}factor\left(Holidays\right) \end{array}$$ (2)

## Results

**Fig 1:**
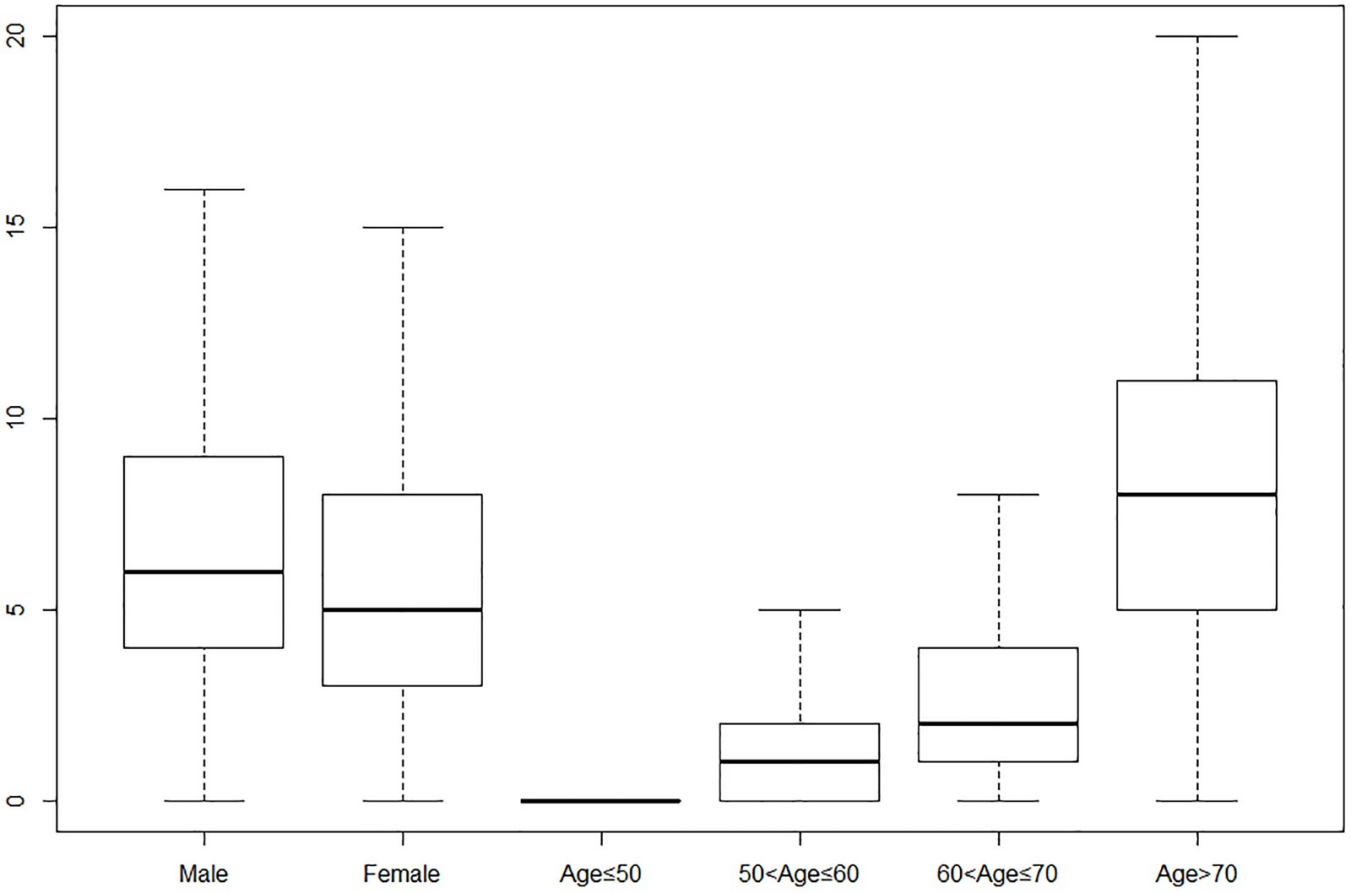
Boxplot of daily AECOPD hospitalization.

Descriptive statistical results of daily ambient air pollution and meteorological factors from 2014 to 2018 were showed in Tables 1 and 2. Table 3 demonstrated the population amount of AECOPD based on the stratification of age and gender. Fig 1 shows boxplot of daily AECOPD hospitalization during research period. It was found that the amount of group age≤50 was less than the other group. The amount of male group for AECOPD hospitalization was larger than that among female group.

**Table 1:** Descriptive statistics for daily air pollution in Shenyang, China from 2014 to 2017.

Variable(μg/m^3^)	X±S	Min	P_25_	P_50_	P_75_	Max	IQR
PM_2.5_	60±50	4	30	46	77	848	47
PM_10_	102±67	8	59	87	126	912	67
SO_2_	52±55	3	15	133	146	333	131
NO_2_	43±17	13	30	39	52	125	22
O_3_	58±33	9	32	53	78	218	46

Min: minimum; Max: maximum; IQR: inter quartile range; X: mean value; S: standard deviation

**Table 2:** Descriptive statistics for daily meteorological factors in Shenyang, China from 2014 to 2017.

Meteorological Factors	X±S	Min	P_25_	P_50_	P_75_	Max	IQR
Temperature (°C)	9±13	-25	-3	11	21	30	24
Humidity (%)	46±26	2	17	51	67	98	50
Pressure (hPa)	1017±71	987	1008	1016	1025	1041	17
Wind Speed(Km/h)	8±3	2	6	8	10	26	4

Min: minimum; Max: maximum; IQR: inter quartile range; X: mean value; S: standard deviation

**Table 3:** The amount of AECOPD hospitalization based on the stratification of age and gender.

AECOPD		
**Total**		17592
**Gender**	Male	9196
	Female	8396
**Age(X±S)**	age≤50	265
	50<age≤60	1709
	60<age≤70	3742
	Age>70	11876

**Fig 2:**
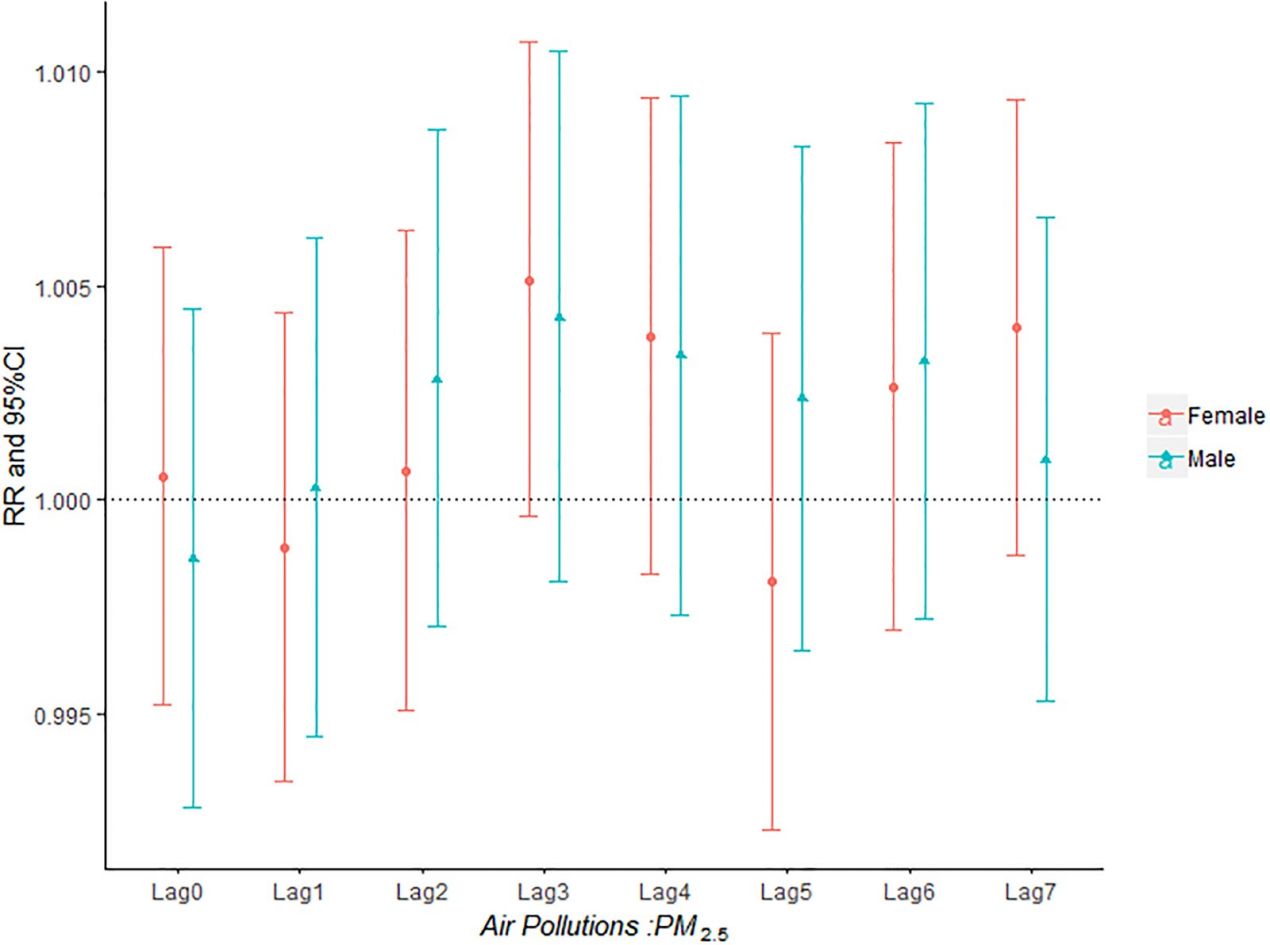
The effect of PM_2.5_ on the relative risk of AECOPD hospitalization based on the stratification of gender.

**Fig 3:**
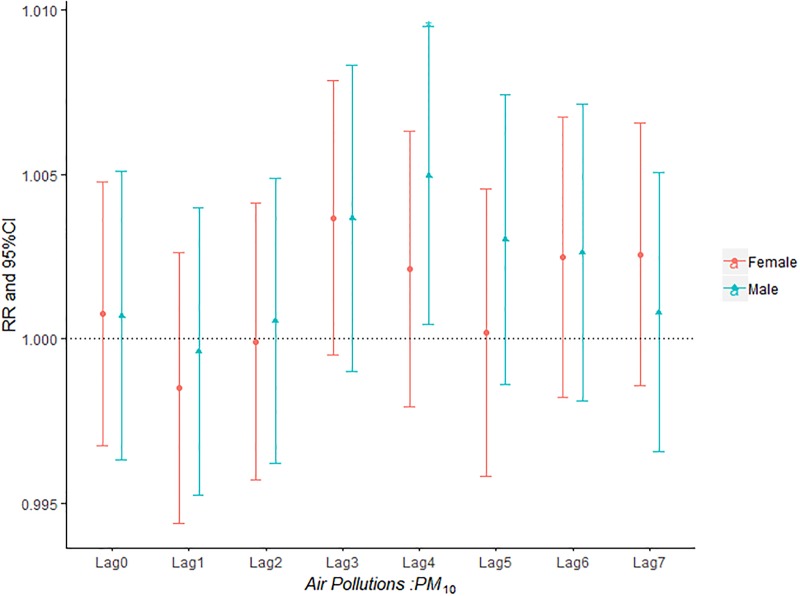
The effect of PM_10_ on the relative risk of AECOPD based on the stratification of gender.

**Fig 4:**
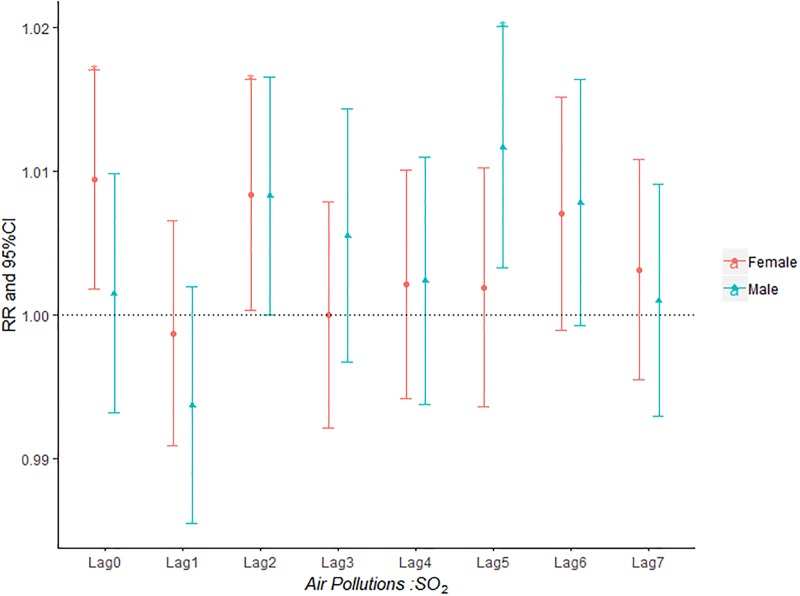
The effect of SO_2_ on the relative risk of AECOPD based on the stratification of gender.

**Fig 5:**
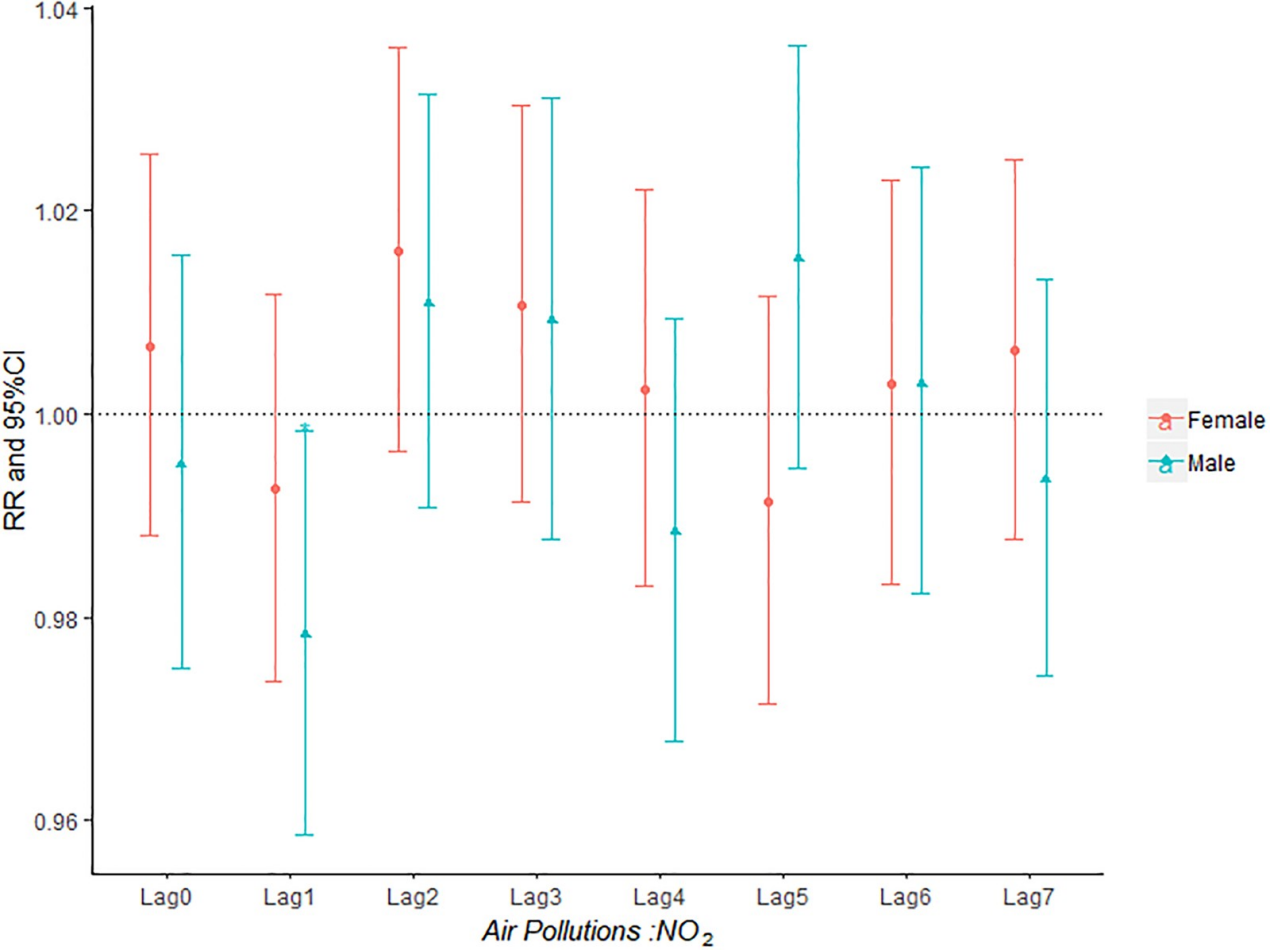
The effect of NO_2_ on the relative risk of AECOPD based on the stratification of gender.

**Fig 6:**
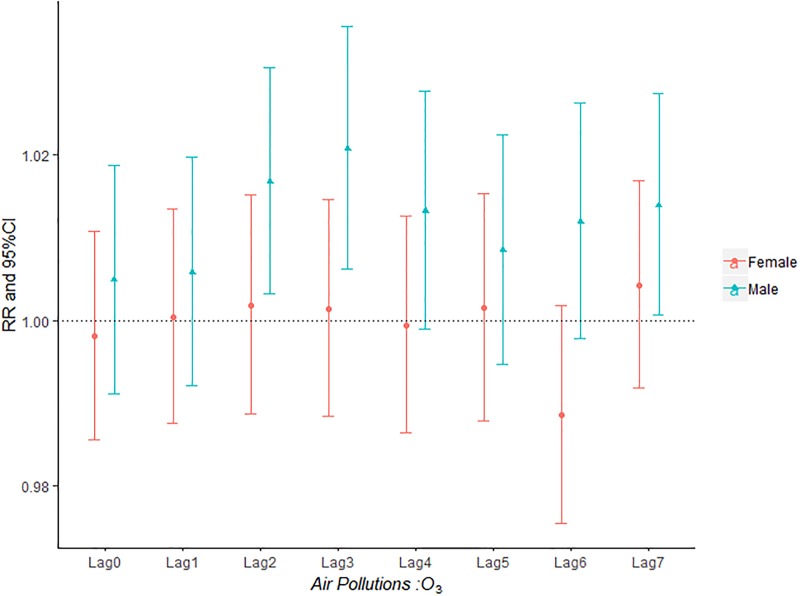
The effect of O_3_ on the relative risk of AECOPD based on the stratification of gender.

The influence of PM_2.5_, PM_10_, SO_2_, NO_2_ and O_3_ on the relative risk of AECOPD hospitalization based on the stratification of gender was shown in Figs 2–6. It was seen from Figs 2 and 3 that there was an obvious lag effect, which was that PM_2.5_ and PM_10_ had the highest impact on AECOPD admission on lag3 and lag4 compared to the other lag days.

Fig 4 shown that both male group and female group was influenced by SO_2_ concentration on the day of admission and female group was more sensitive to SO_2_ than male group. The effect of ozone on admission rate in male group was higher than that in female group (Fig 6).

**Fig 7:**
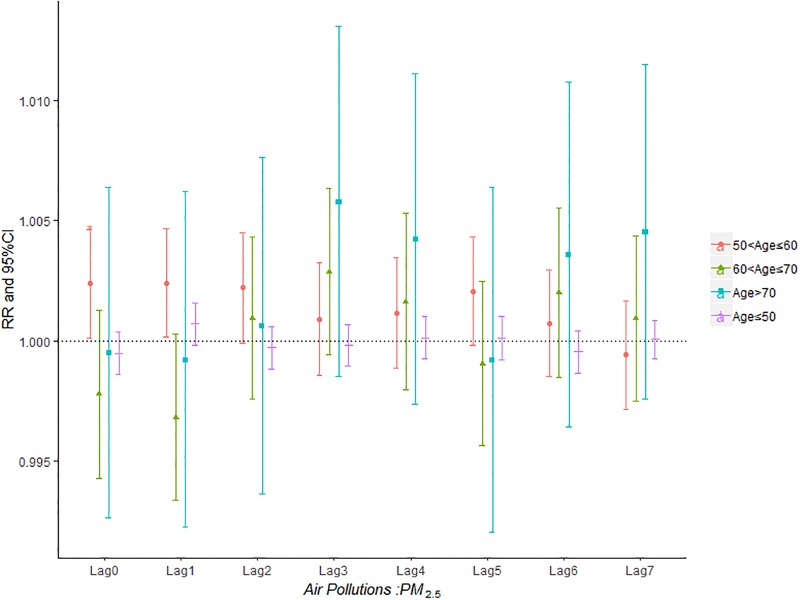
The effect of PM_2.5_ on the relative risk of AECOPD based on the stratification of age.

**Fig 8:**
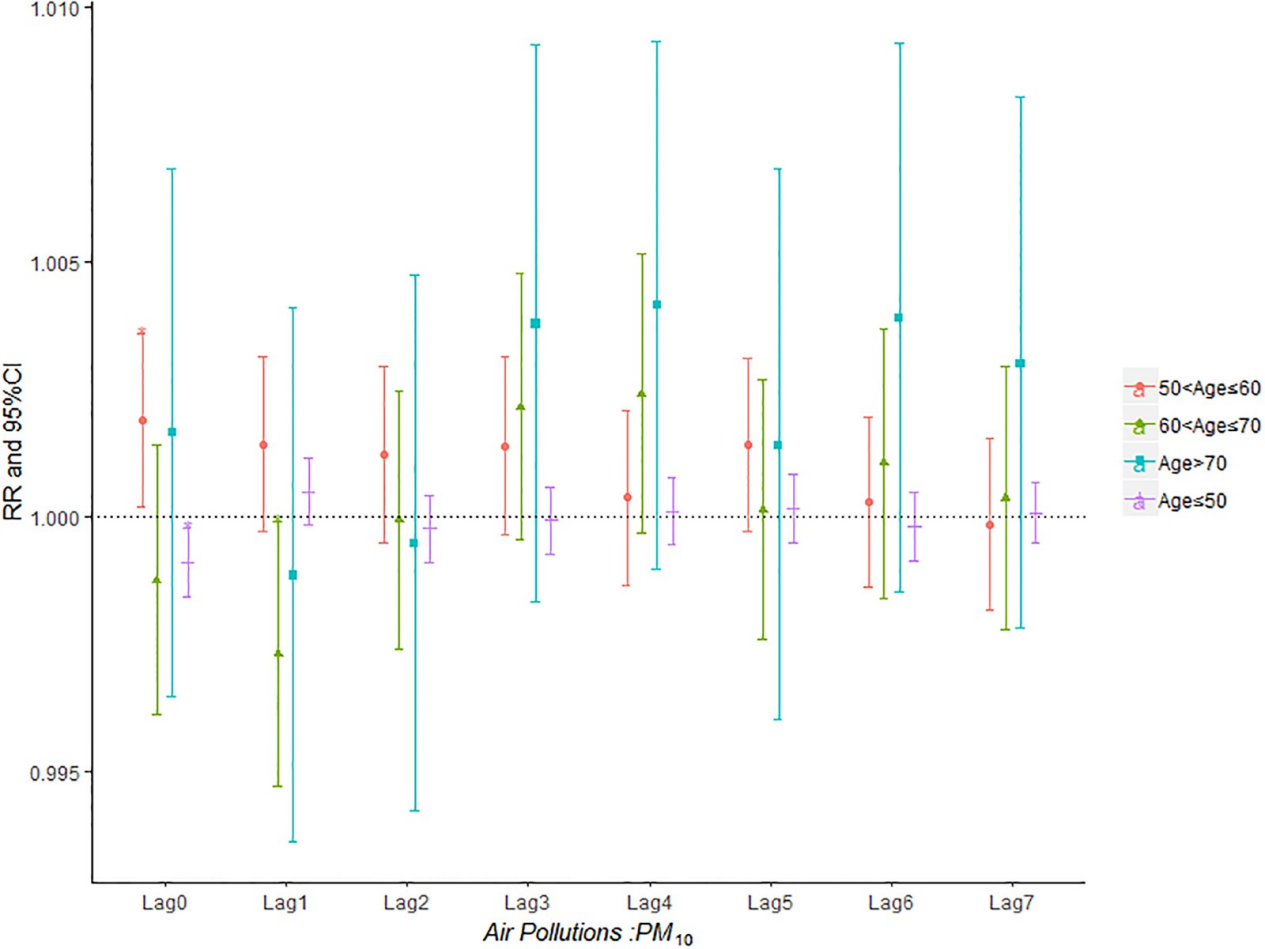
The effect of PM_10_ on the relative risk of AECOPD based on the stratification of age.

**Fig 9:**
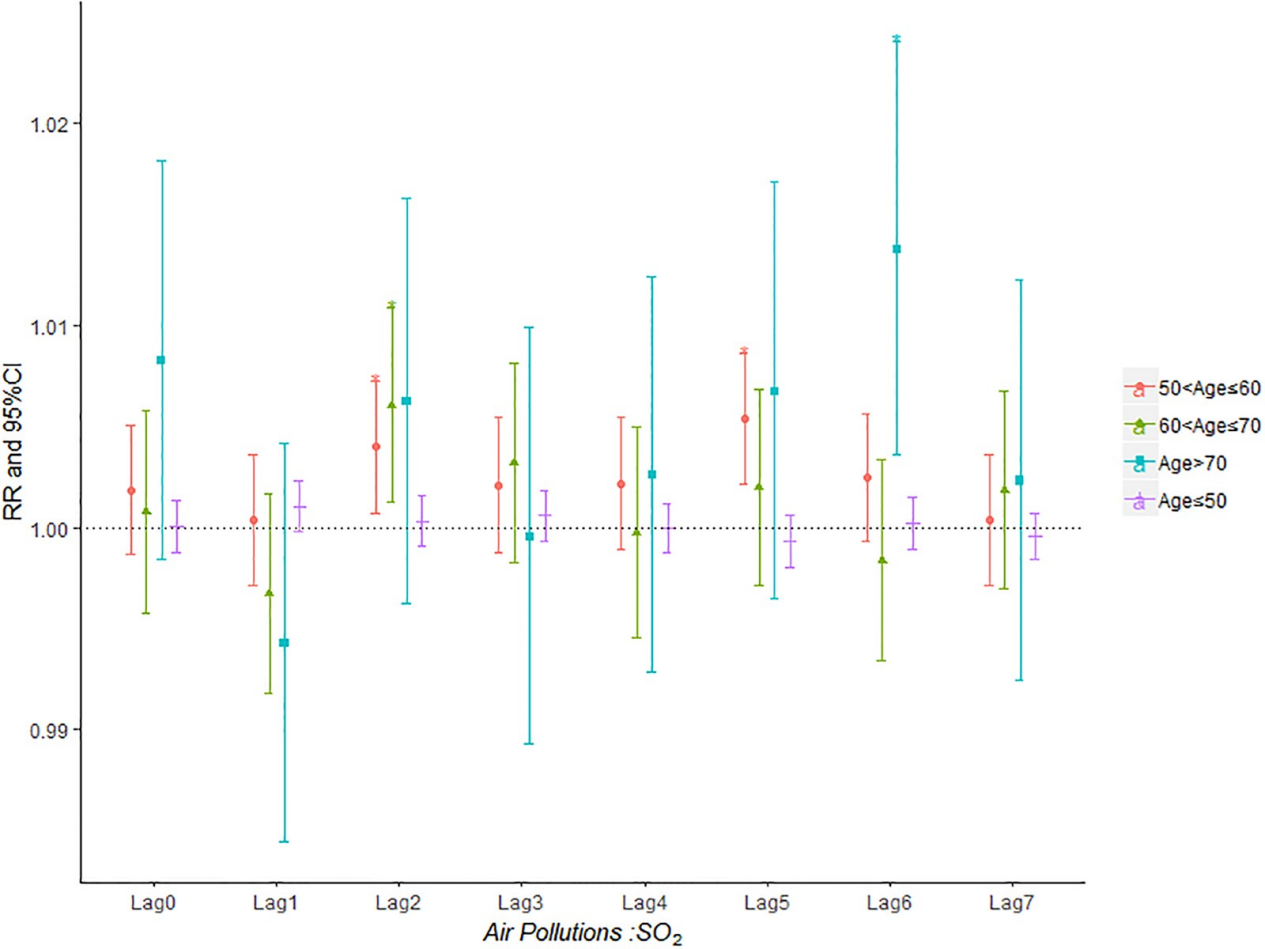
The effect of SO_2_ on the relative risk of AECOPD based on the stratification of age.

**Fig 10:**
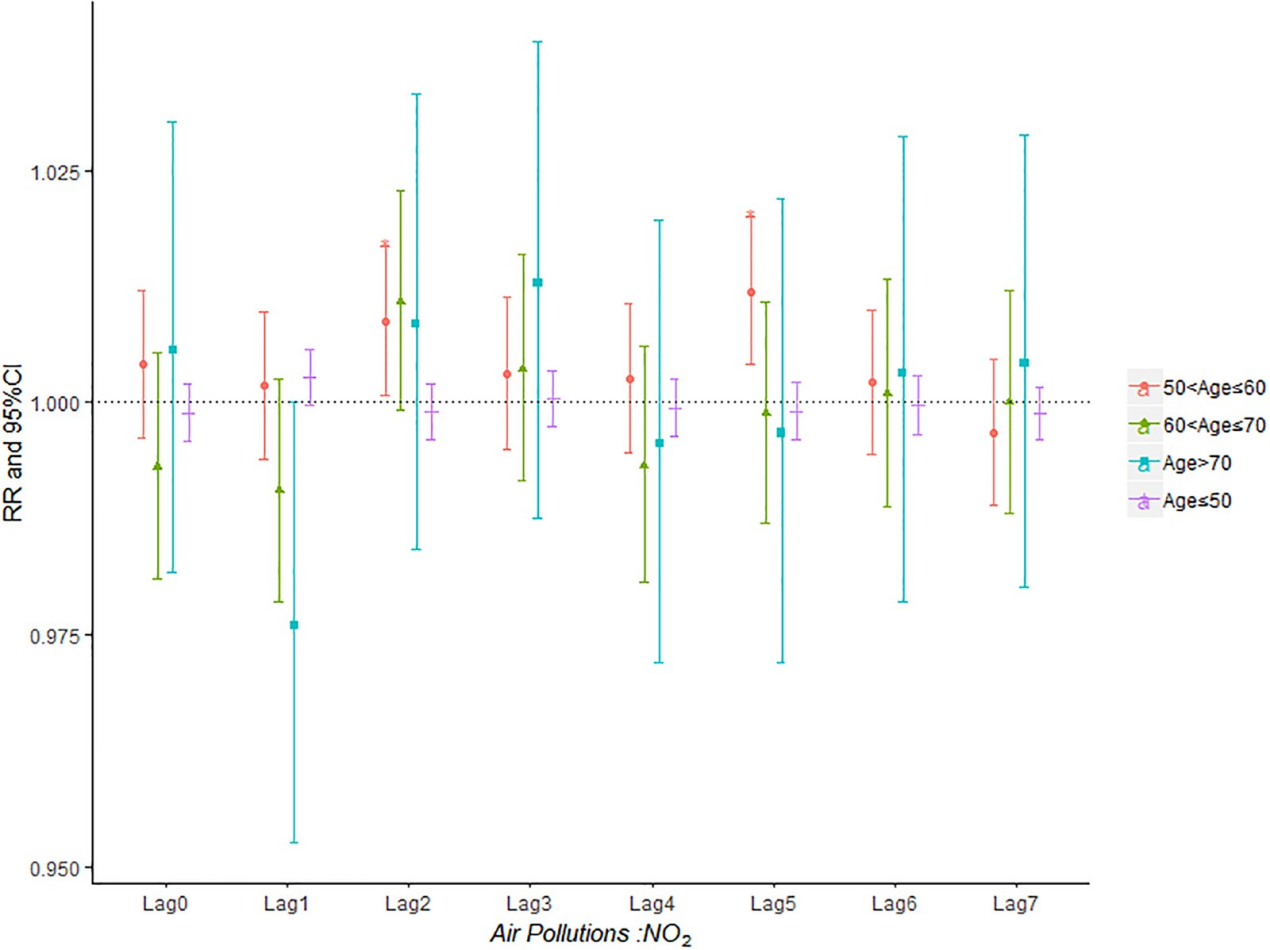
The effect of NO_2_ on the relative risk of AECOPD based on the stratification of gender.

**Fig 11:**
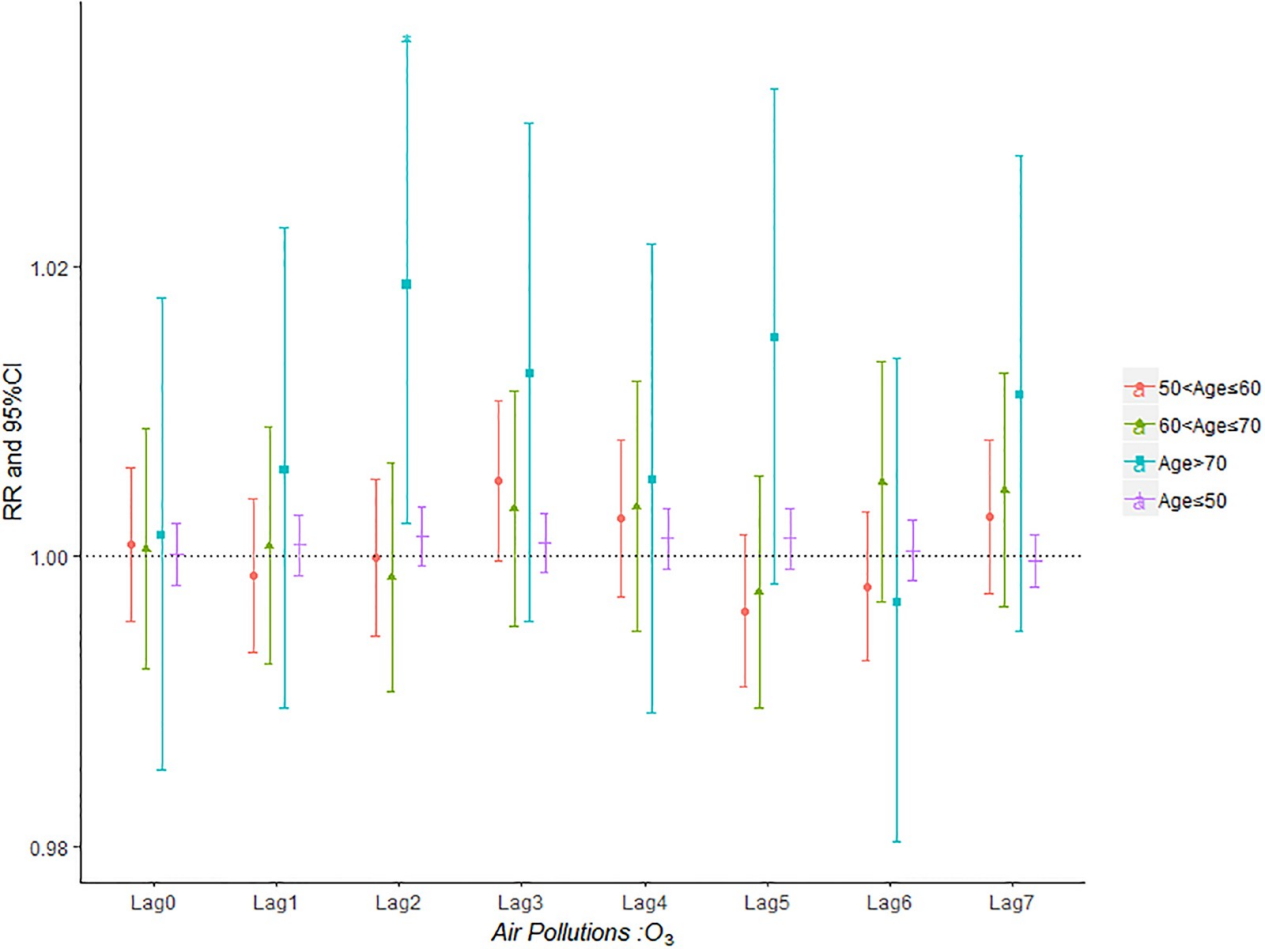
The effect of O_3_ on the relative risk of AECOPD based on the stratification of gender.

Figs 7–11 demonstrated the effect of air pollution on AECOPD based on the stratification of age. Ambient air pollution had a weak influence on age≤50 group (Figs 7–11). Optimal delay date for PM_2.5_ to 60<age≤70 group and age>70 group both appeared on lag3 (Fig 7). PM_10_ could exacerbate 50<age≤60 group hospitalization risk on lag0-lag6 (Fig 8). The risk due to exposure to SO_2_ on lag0, lag2, lag4-6 in the age>70 group was greater than among the other age group (Fig 9). The change in risk by age group due to O_3_ is displayed in Fig 11, revealing that the risk among the age>70 group on lag2 was higher than that among the other three group.

**Fig 12:**
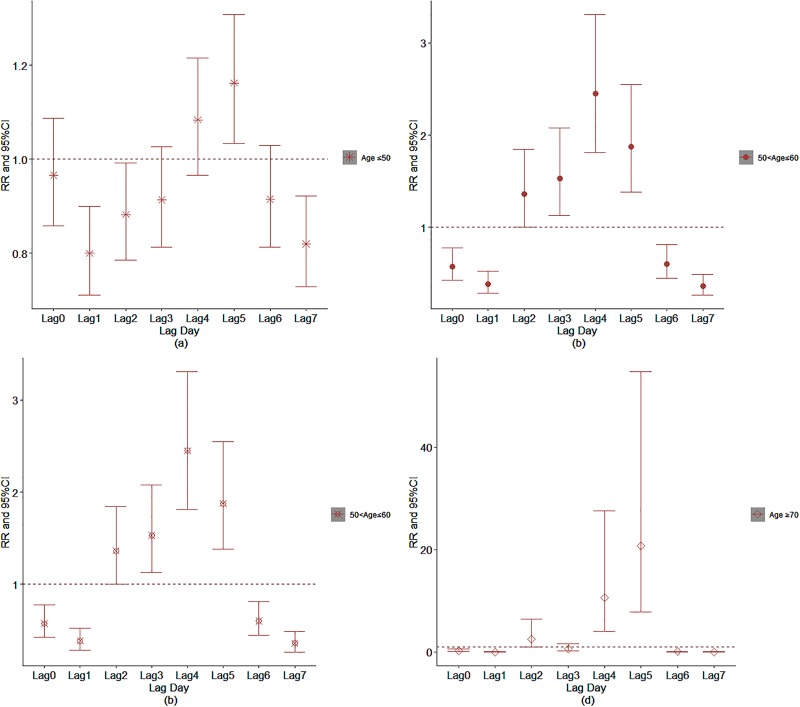
The effect of daily relative humidity on the AECOPD hospitalization based on the stratification of age; (a: Age≤50; b: 50<age≤60; c: 60<age≤70; d: Age>70).

**Fig 13:**
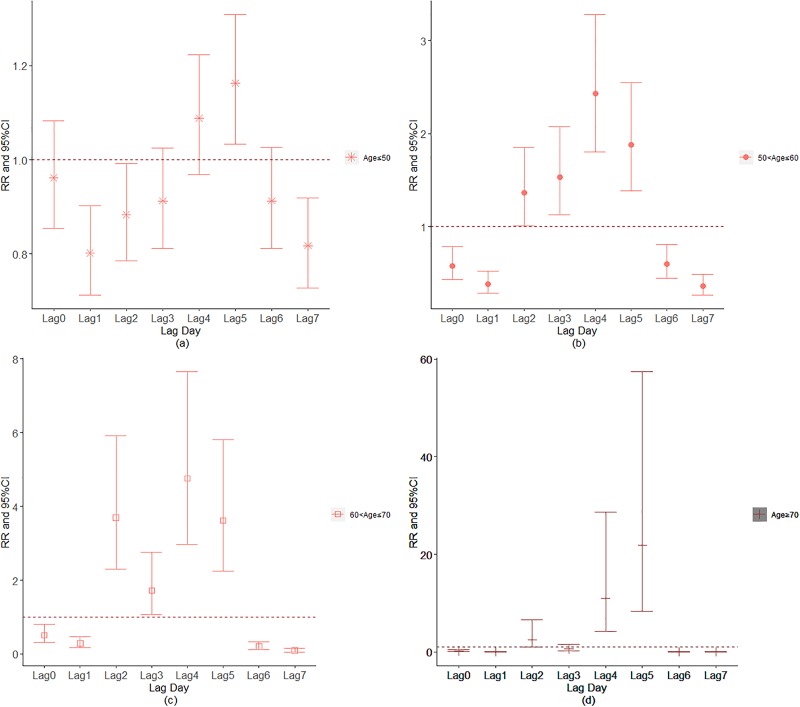
The effect of daily average temperature on the AECOPD hospitalization based on the stratification of age (a: Age≤50; b: 50< age≤60; c: 60< age≤70; d: Age>70).

**Fig 14:**
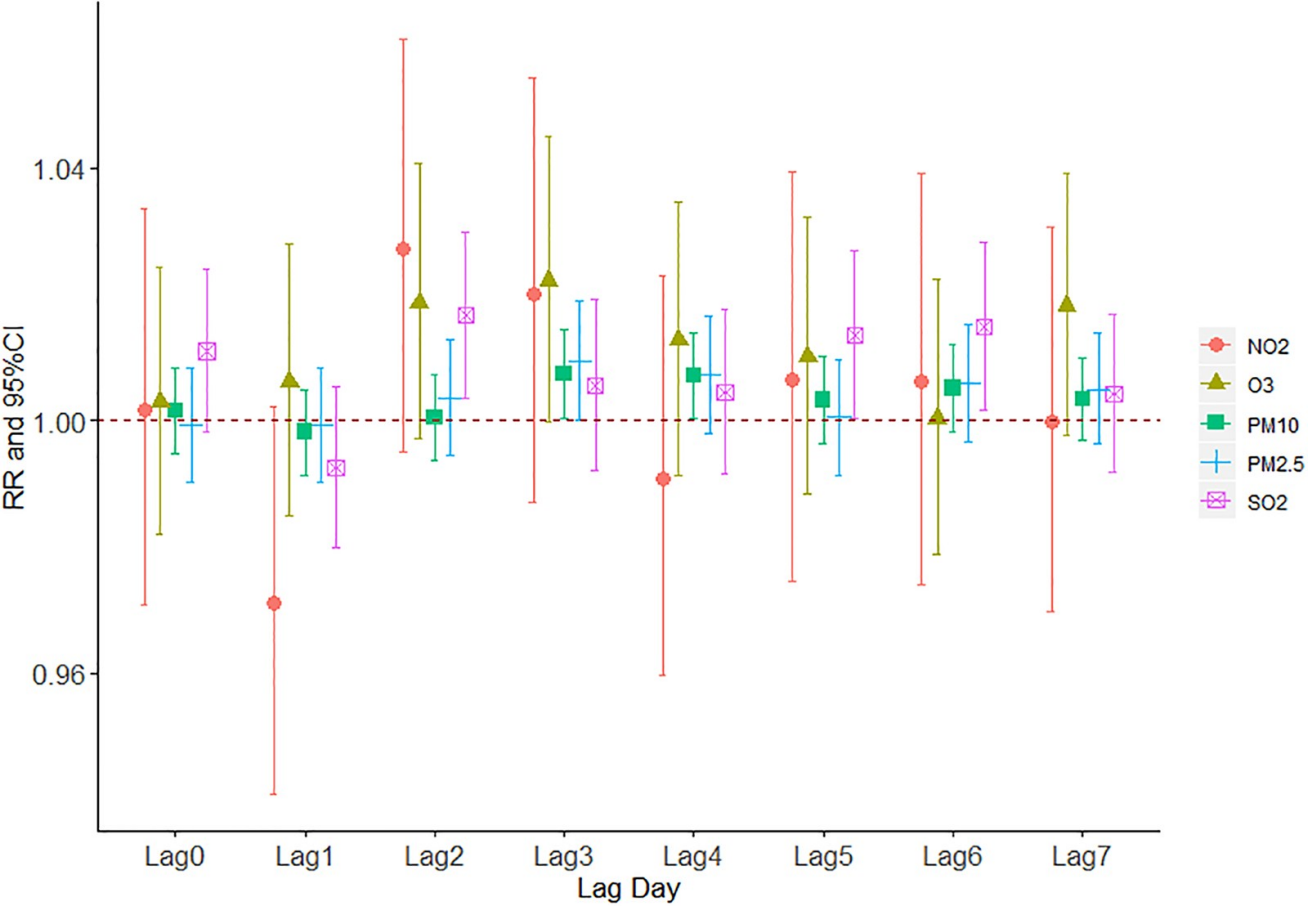
The effect of ambient air pollution on the total AECOPD hospitalization.

The effect of daily relative humidity on the AECOPD hospitalization based on the stratification of age was displayed in Fig 12. It was found that the optimal lag day for daily relative humidity to age≤50 group, 50<age≤60, 60<age≤70 group and age>70 group was on lag5, lag4, lag4 and lag5, respectively. Similarly, the optimal lag day for age≤50 group, 50<age≤60, 60<age≤70 group and age>70 group under the influence of daily average temperature occurred on lag5, lag4, lag4 and lag5, respectively(Fig 13). Fig 14 demonstrated the effect of ambient air pollution on the total AECOPD hospitalization.

## Discussions

Chronic obstructive pulmonary disease (COPD) has become one of the main contributors to the global burden of disease. It was reported that COPD was the third leading cause of death in China[16]. This paper purposed to investigate the relationship between hospitalization of acute exacerbations of COPD and ambient air pollution, daily average temperature and daily average pressure.

It was found that there was a delayed effect between the hospitalization of AECOPD and ambient gaseous pollution (PM_2.5_ and PM_10_). Our results are consistent with previous studies. One research conducted in China, found that the largest effect of PM_2.5_ and PM_10_ on the AECOPD admission was observed on lag12 (RR, 1.068; 95%CI, 1.017–1.121) and lag10 (RR, 1.031; 95%CI, 1.002–1.060), respectively[3]. Additionally, there was a statistical significance between AECOPD hospitalization and the particulate matter levels of three days before hospitalization[17]. One possible reason, which could be account for this phenomenon, was that particulate matter was attached to the respiratory mucosa or deposited in the bottom of lung after it entering human body, thus which induced pneumonia response and oxidative stress response. This process leads to a delayed effect [13]. When PM entered human’s respiratory tract, its metal component such Zn^2+^, Cu^2+^ and etc. can increase the production of reactive oxygen species (ROS), which damages the dynamic balance of ROS and leads to the imbalance of oxidation-antioxidant mechanism, which could aggravate the symptom of COPD[18]. Exposure to fine particulate matter could cause infiltration of inflammatory cell and increase the release of inflammatory factors such as interleukin, which damage people’s respiratory tract and lung. Suspension of PM_2.5_ was intranasally instilled to rats, it generated higher level of interleukin-6 and tumor necrosis factor-α and activated the body’s immune response. When the human body is exposed to ambient SO_2_, due to its good water-solubility, part of SO_2_ dissolved directly in the mucous membrane of the respiratory tract to form SO_3_^2-^, resulting in toxic effects on the respiratory system of the human body. The other part entered the bloodstream and dissolved directly in the body to form SO_3_^2-^, which acted as a toxic agent[19]. The elderly (≥70 years) showed that the most susceptible group to the effect of ambient air pollution, which could be explained that the elderly’s respiratory fiber villi clearance ability and immunity decreased[20].

In this paper, we investigated the effect of daily average temperature and humidity on the hospitalization of AECOPD. We found that daily average temperature had an adverse effect on acute admission of COPD, which seems to be in accord with recent studies indicating that the aged was more susceptible with RR values of 1.048 (95%CI: 1.029–1.066) in cold season[21]. A published report demonstrated that low temperature could enhance exacerbate COPD symptom, indoor temperature was supposed to keep at least on average at 18.2°C, while for people in Shenyang, located in northeast China (123.38E, 41.8N), more attention should paid to low temperature[22]. Another research showed that a 1°C decrease in temperature was linked with a 0.8% increase for AECOPD admission on event-days (95% CI: 1.015–1.138). Moreover, with a 5°C decrease in average temperature, the low temperature (28-day average temperature) was a long-term effect on AECOPD [23]. Our results confirm the association between humidity and AECOPD admission. A population-based study in a metropolitan area found that hospital admissions increased by 5.04%, for per degree Celsius decrease in average weekly temperature[24].

## Conclusions

The present study was designed to determine the effect of air pollution (PM_2.5_, PM_10_, SO_2_, NO_2_ and O_3_), relative humidity and temperature on the hospitalization of acute exacerbation of COPD. The findings of this research provide insights for that air pollution, relative humidity and temperature can increase the risk of admission for acute exacerbation of COPD. The findings of this investigation complement those of earlier studies, especially in Shenyang.

The limitations of this paper are as followings. We didn’t consider that the time of hospitalization visits was on daytime or nighttime for that these data were not available. Seasonal factor was not considered in this paper. Our future work is to investigate the long-term effect of seasonal factor on the AECOPD admission.

## Supporting information

S1 File This is air pollution data in this study. (XLSX)

S2 File This is meteorology data in this study. (XLSX)

S3 File This is the visual code in R language. (TXT)

## Abbreviations

AECOPD: acute exacerbation of chronic obstructive pulmonary disease
CI: confidence interval
NO_2_: nitrogen dioxide
O_3_: Ozone
PM: particulate matter
RR: relative risk
SO_2_: sulfur dioxide

## References

[1] Gruzieva O, Xu CJ, Yousefi P, Relton C, Merid SK, Breton CV, et al Prenatal Particulate Air Pollution and DNA Methylation in Newborns: An Epigenome-Wide Meta-Analysis. Environ Health Perspect. 2019;127(5):57012 10.1289/EHP4522 31148503 PMC6792178

[2] Ho SC, Chuang KJ, Lee KY, Chen JK, Wu SM, Chen TT, et al Chronic obstructive pulmonary disease patients have a higher risk of occurrence of pneumonia by air pollution. SCI TOTAL ENVIRON. 2019;677:524–9. 10.1016/j.scitotenv.2019.04.358 31063895

[3] Xie J, Teng J, Fan Y, Xie R, Shen A. The short-term effects of air pollutants on hospitalizations for respiratory disease in Hefei, China. INT J BIOMETEOROL. 2019;63(3):315–26. 10.1007/s00484-018-01665-y 30680626

[4] Sun X, Wei H, Young DE, Bein KJ, Smiley-Jewell SM, Zhang Q, et al Differential pulmonary effects of wintertime California and China particulate matter in healthy young mice. TOXICOL LETT.2017;278:1–8. 10.1016/j.toxlet.2017.07.853 28698096 PMC5572813

[5] Jin Y, Wu W, Zhang W, Zhao Y, Wu Y, Ge G, et al Involvement of EGF receptor signaling and NLRP12 inflammasome in fine particulate matter-induced lung inflammation in mice. ENVIRON TOXICOL.2017;32(4):1121–34. 10.1002/tox.22308 27377055

[6] Wigenstam E, Elfsmark L, Bucht A, Jonasson S. Inhaled sulfur dioxide causes pulmonary and systemic inflammation leading to fibrotic respiratory disease in a rat model of chemical-induced lung injury. TOXICOLOGY. 2016;368:28–36. 10.1016/j.tox.2016.08.018 27565714

[7] Han M, Ji X, Li G, Sang N. NO2 inhalation enhances asthma susceptibility in a rat model. Environ Sci Pollut Res Int. 2017;24(36):27843–54. 10.1007/s11356-017-0402-7 28986735

[8] Rocks N, Vanwinge C, Radermecker C, Blacher S, Gilles C, Maree R, et al Ozone-primed neutrophils promote early steps of tumour cell metastasis to lungs by enhancing their NET production. THORAX. 2019;74(8):768–79. 10.1136/thoraxjnl-2018-211990 31142617

[9] Barbone F, Catelan D, Pistelli R, Accetta G, Grechi D, Rusconi F, et al A Panel Study on Lung Function and Bronchial Inflammation among Children Exposed to Ambient SO2 from an Oil Refinery. INT J ENV RES PUB HE. 2019;16(6):1057. 10.3390/ijerph16061057 PMC6466338 30909566

[10] Jo EJ, Lee WS, Jo HY, Kim CH, Eom JS, Mok JH, et al Effects of particulate matter on respiratory disease and the impact of meteorological factors in Busan, Korea. Respir Med.2017;124:79–87. 10.1016/j.rmed.2017.02.010 28284326

[11] Tasci SS, Kavalci C, Kayipmaz AE. Relationship of Meteorological and Air Pollution Parameters with Pneumonia in Elderly Patients. EMERG MED INT.;2018:4183203 10.1155/2018/4183203 29755789 PMC5884022

[12] Sun S, Laden F, Hart JE, Qiu H, Wang Y, Wong CM, et al Seasonal temperature variability and emergency hospital admissions for respiratory diseases: a population-based cohort study. THORAX.2018;73(10):951–8. 10.1136/thoraxjnl-2017-211333 29622691

[13] Dehghan A, Khanjani N, Bahrampour A, Goudarzi G, Yunesian M. The relation between air pollution and respiratory deaths in Tehran, Iran- using generalized additive models. BMC PULM MED.2018;18(1):49 10.1186/s12890-018-0613-9 29558916 PMC5859399

[14] Chen C, Wang X, Lv C, Li W, Ma D, Zhang Q, et al The effect of air pollution on hospitalization of individuals with respiratory and cardiovascular diseases in Jinan, China. MEDICINE. 2019;98(22):e15634 10.1097/MD.0000000000015634 31145279 PMC6708625

[15] Tian Y, Xiang X, Juan J, Song J, Cao Y, Huang C, et al Short-term effects of ambient fine particulate matter pollution on hospital visits for chronic obstructive pulmonary disease in Beijing, China. Environ Health. 2018;17(1):21 10.1186/s12940-018-0369-y 29482552 PMC6389038

[16] Zhu B, Wang Y, Ming J, Chen W, Zhang L. Disease burden of COPD in China: a systematic review. Int J Chron Obstruct Pulmon Dis. 2018;13:1353–64. 10.2147/COPD.S161555 29731623 PMC5927339

[17] Choi J, Oh JY, Lee YS, Min KH, Hur GY, Lee SY, et al Harmful impact of air pollution on severe acute exacerbation of chronic obstructive pulmonary disease: particulate matter is hazardous. Int J Chron Obstruct Pulmon Dis. 2018;13:1053–9. 10.2147/COPD.S156617 29681728 PMC5881527

[18] Waterston A, Castillo J, Olivas M, Hasson A, Dejean L. PM2.5 Exposure and ROS Production in NR8383 Rat Alveolar Macrophages. BIOPHYS J. 2018;1141(3):334A.

[19] Vepkhvadze N, Kiladze N, Khorbaladze M, Kochoradze T, Kugoti I. IMPACT OF SULPHUR DIOXIDE ON THE RESPIRATORY SYSTEM OF TBILISI POPULATION. Georgian Med News. 2017(265):1114–9. 28574394

[20] Phosri A, Ueda K, Phung V, Tawatsupa B, Honda A, Takano H. Effects of ambient air pollution on daily hospital admissions for respiratory and cardiovascular diseases in Bangkok, Thailand. SCI TOTAL ENVIRON. 2019;651(Pt 1):1144–53. 10.1016/j.scitotenv.2018.09.183 30360246

[21] Ma Y, Zhao Y, Zhou J, Jiang Y, Yang S, Yu Z. The relationship between diurnal temperature range and COPD hospital admissions in Changchun, China. ENVIRON SCI POLLUT R. 2018;25(18):17942–9. 10.1007/s11356-018-2013-3 29680890

[22] Mu Z, Chen PL, Geng FH, Ren L, Gu WC, Ma JY, et al Synergistic effects of temperature and humidity on the symptoms of COPD patients. INT J BIOMETEOROL. 2017;61(11):1919–25. 10.1007/s00484-017-1379-0 28567499

[23] Tseng CM, Chen YT, Ou SM, Hsiao YH, Li SY, Wang SJ, et al The effect of cold temperature on increased exacerbation of chronic obstructive pulmonary disease: a nationwide study. PLOS ONE. 2013;8(3):e57066 10.1371/journal.pone.0057066 23554858 PMC3598847

[24] Almagro P, Hernandez C, Martinez-Cambor P, Tresserras R, Escarrabill J. Seasonality, ambient temperatures and hospitalizations for acute exacerbation of COPD: a population-based study in a metropolitan area. INT J CHRONIC OBSTR. 2015;10:899–908. 10.2147/COPD.S75710 PMC4431472 26056439

